# MAMs Protect Against Ectopic Fat Deposition and Lipid-Related Kidney Damage in DN Patients

**DOI:** 10.3389/fendo.2021.609580

**Published:** 2021-02-19

**Authors:** Ming Yang, Yachun Han, Shilu Luo, Xiaofen Xiong, Xuejing Zhu, Hao Zhao, Na Jiang, Ying Xiao, Ling Wei, Chenrui Li, Jinfei Yang, Lin Sun

**Affiliations:** Department of Nephrology, The Second Xiangya Hospital of Central South University, Hunan Key Laboratory of Kidney Disease and Blood Purification, Changsha, China

**Keywords:** mitochondria-associated ER membranes (MAMs), ectopic fat deposition, diabetic nephropathy (DN), lipid metabolism, mitochondria

## Abstract

Ectopic fat deposition (EFD) in the kidney plays a key role in the development of diabetic nephropathy (DN). Mitochondria-associated ER membranes (MAMs) are structures that connect to the endoplasmic reticulum (ER) and are involved in lipid metabolism. However, there are few studies on MAMs in the field of kidney disease, and the relationship between EFD and MAMs in DN is still unclear. In this study, increased EFD in the kidneys of DN patients was observed, and analysis showed that the degree of EFD was positively correlated with renal damage. Then, the MAMs were quantified by an *in situ* proximity ligation assay (PLA). The MAMs in the kidneys were found to gradually decrease through the different stages of DN, while the expression of ADRP (a marker of lipid droplets) and tubulointerstitial damage increased. Moreover, correlation analysis showed that the MAMs were negatively correlated with serum lipid levels, the EFD in the kidney and renal damage. Finally, we observed decreased expression of MAM-control proteins (DsbA-L, PACS-2, and MFN-2) in different stages of DN and they were associated with lipid deposition and renal damage. These data showed that the destruction of MAMs in DN might be the cause of EFD and interstitial damage in the kidney.

## Introduction

Diabetic nephropathy (DN) is the leading cause of end-stage renal disease (ESRD), and lipid metabolism is disorders are an important clinical manifestation of DN (1, 2). Based on the histopathology observed in kidney biopsies, DN is classified into four types: class I, the glomerular basement membrane is thickened, with only mild and nonspecific changes visible under light microscopy; class II, mild (IIa) or severe (IIb) glomerular mesangial expansion; class III, there is at least one nodular mesangial matrix (Kimmelstiel–Wilson lesions); and class IV, there is more than 50% glomerular sclerosis. With the aggravation of renal pathology, clinical manifestations such as proteinuria will be further aggravated (3). Recent studies have shown that high glucose induces lipid deposition in the kidney in animals (4), and lipid deposition in non-adipose tissues such as the liver and kidney is called ectopic fat deposition (EFD) (5). Triglyceride (TG) and cholesterol (CHOL) accumulation in the kidneys promotes the expression of sterol regulatory element-binding protein-1 and aggravates the pathological changes in the kidney (6). Furthermore, lipid accumulation in renal tubular epithelial cells also leads to increased production of reactive oxygen species (ROS) and inflammatory cytokines, which further aggravate the kidney injury (7, 8). These findings suggest that EFD plays an important role in the development of diabetic nephropathy. Currently, many factors are known to be involved in the development of lipid deposition in the kidney, such as abnormal oxidation of fatty acids (9), inflammation (10), and angiotensin II (11). However, the specific mechanism of renal lipid deposition needs further study.

Mitochondria-associated ER membranes (MAMs) are the structure that comprised of the endoplasmic reticulum (ER) subdomain, part of the outer mitochondrial membrane (OMM) and proteins (12). MAMs have been shown to be involved in a variety of cellular physiological activities, and lipid metabolism is one of its most important functions (13). Enzymes involved in lipid metabolism, such as dicylgycerol acyltransferase 2 (DGAT2), phosphatidylserine synthase (PSS), and acyl coenzyme A-cholesterol acyltransferase (ACAT), were found to be located on MAMs (12), and their structural and functional abnormalities are closely related to the development of diabetes, especially insulin resistance (14, 15). In previous studies, we also proved that abnormal MAMs were associated with apoptosis in STZ-induced diabetic DN mice and in HK-2 cells after a high glucose intervention (16). This evidence indicates that MAMs play an important role in the pathological process of DN.

In summary, we investigated the integrity of MAMs in the kidneys of DN patients, and correlations between MAMs and renal lipid deposition and clinical characteristics were established. We noted that the integrity of MAMs was notably decreased in DN patients compared with controls, and the decreased MAMs were associated with increased lipid deposition in the kidney, serum lipid levels, and renal tubulointerstitial damage. This data suggests that MAMs may be potential therapeutic targets for alleviating lipid renal damage in DN.

## Materials and Methods

### Participants and Study Design

We recruited 10 patients with glomerular minor lesions (GMLs) as controls and 30 patients with different stages of diabetic nephropathy who all provided informed consent for inclusion in this study. This study was conducted in accordance with the ethical guidelines and was approved by the institutional review board. The patients with DN were first diagnosed by renal biopsy at the Department of Nephrology, The Second Xiangya Hospital of Central South University. The DN patients were treated with insulin to control their blood glucose, but patients who used lipid-lowering medicine or RAS inhibitors before the renal biopsy or had other diseases, such as urinary tract infections or neoplastic, cardiovascular, hepatic, renal, lung or neuroendocrine diseases were excluded from the group. According to the 2010 pathologic classification of DN, the patients who had renal biopsy were divided into class IIa, class IIb, and class III groups. There were 10 patients with class IIa DN (5 males and 5 females), aged 37–52 years (mean: 46 ± 7.32 years); the class IIb group included 10 patients (6 males and 4 females), aged 42–64 years (mean: 55 ± 8.64 years); and class III also included 10 patients (4 males and 6 females), aged 44–63 years (mean: 53 ± 9.66 years). The control group included patients with nondiabetic nephropathy, such as glomerular minor lesions (GMLs) (n = 10, 4 males and 6 females), aged 37–51 years (mean: 43 ± 8.72 years). Adrenocortical hormone and immunosuppression drugs were not used during the course of the patient’s treatment.

### Biochemical Analysis of Blood and Urine

Standard automated enzymatic methods (Hitachi 912 automated analyzer) were used to analyze the liver function index, such as total proteins (TP) and albumin (Alb), renal function index, such as creatinine (Cr), blood urea nitrogen (BUN), and uric acid (UA), serum lipid levels such as total cholesterol (TC), high-density lipoprotein cholesterol (HDL-C), low-density lipoprotein cholesterol (LDL-C), and triglyceride, as described previously (17, 18). The automated colorimetric method was used to determine the content of N-acetyl-β-D-glucosaminidase (β-NAG).

### Renal Morphological Analysis

The kidney tissues from the renal biopsy of the diabetic nephropathy group and the control group were fixed in 10% moderate formalin and then embedded in paraffin. Hematoxylin-eosin (H&E), periodic acid Schiff (PAS), and periodic acid-silver metheramine (PASM) staining were performed on 4 μm thick paraffin sections to observe the pathological changes in the kidney during diabetic nephropathy. The tubulointerstitial injury score was evaluated as described previously (17).

### Immunohistochemistry

The renal tissue slides were deparaffinized with xylene and then dehydrated with a gradient concentration of alcohol. The slides were immersed in antigen repair solution for 8 min at high heat and 20 min at medium heat for antigen repair, and then naturally cooled to room temperature. After blocking, the slides were then incubated with primary antibodies against DsbA-L, MFN-2, and PACS-2 overnight at 4°C. After rewarming, the secondary antibody and diaminobenzidine (DAB) substrate were incubated sequentially. After hematoxylin counterstaining and dehydration, the slides were sealed with neutral resin and then observed and photographed under a Nikon Eclipse 50i microscope.

### In Situ Proximity Ligation Assay

The integrity of the MAMs was assessed by a Duolink II *in situ* proximity ligation assay (PLA) (Olink Bioscience), which enables the interaction between protein-protein interactions to be detected, visualized, and quantified. The paraffin-embedded kidney sections were treated with dewaxing, rehydration, and antigen retrieval, and then incubated overnight at 4°C with anti-voltage-dependent anion channel 1 (VDAC1) antibody (Proteintech, 66345-1-Ig, 1:500) and anti-IP3R1 antibody (Proteintech, 19962-1-AP, 1:200), which are the conjugated proteins on the MAMs, using the methods previously described (16). The sections were incubated with two PLA probes for rabbits and mice for 1 hr, and ligation and amplification were subsequently performed in the presence of polymerase. After nucleic acid dye counterstaining and dehydration and sealing with neutral resin, images were obtained under a microscope. The integrity of the MAMs was assessed by ImageJ software, and the IP3R1-VDAC1 dots were quantified and are expressed as dots per nucleus, as described previously (14, 16). The experiments were performed at least three times for each donor, with a minimum of five random fields taken per condition.

### The Mitochondria-Associated Endoplastic Reticulum Membrane Integrity Was Assessed by Transmission Electron Microscopy Analysis

The ER-mitochondrial contacts were assessed by transmission electron microscopy (TEM). The percentage of mitochondrial membrane in contact with the ER within a 50 nm range was measured and normalized to mitochondrial perimeter, as previously reported (14).

### Statistical Analysis

SPSS 20.0 software was used to perform the statistical analyses. The data are expressed as Mean ± SD. One-way ANOVA was used to compare the differences among the groups. The correlation of the two numerical variables was tested by Pearson’s analysis, and the statistically significance was considered when the p value < 0.05.

## Results

### General Characteristics and Laboratory Results of the Diabetic Nephropathy Patients

**Figure 1:**
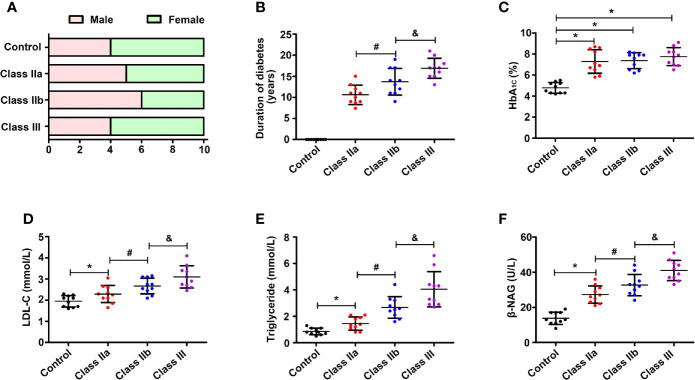
Laboratory results of blood and urine. One-way ANOVA was used to compare the differences among the groups. **(A)** Sex ratio in the control group and DN groups. **(B)** Distribution of duration in the DN groups. **(C)** Distribution of the HbA1c level in different groups. **(D)** Distribution of the LDL-c level in different groups. **(E)** Distribution of the TG level in different groups. **(F)** Distribution of the β-NAG level in different groups. Values are the mean±SD. *p < 0.05 compared with the control group; ^#^p < 0.05 compared with the class IIa group; ^&^p < 0.05 compared with the class IIb group.

The patients were divided into four groups based on the pathological results of the kidney biopsy. As shown in **Figure 1A**, the sex ratios were control (4/6, male/female), class IIa (5/5), class IIb (6/4), and class III (4/6). Compared to the class IIa group, the DN patients in the class IIb group showed longer average duration years, and the disease duration was further extended in the class III group (p < 0.05) (**Figure 1B**). Since the level of HbA1c is an indicator of whether blood glucose is well controlled over a long period of time, TG and LDL-c reflect lipid levels (an important factor in DN progression), and elevated NAG levels represent renal injury. Therefore, these indicators were observed in different stages of DN patients. All groups of DN patients showed significantly increased levels of HbA1c (p < 0.05) compared to the controls (**Figure 1C**). Moreover, higher lipid levels (LDL-c and TG) were noted in the DN patients than in the controls, and the levels were increased in class IIb compared to class IIa, while they were further aggravated in the class III DN group (p < 0.05) (**Figures 1D, E** **)**. The urinary β-NAG levels in the DN groups were notably increased compared with those in the control group (p < 0.05). The levels of β-NAG in the class IIb and class III groups were dramatically higher than those in the class IIa group (p < 0.05) **(** **Figure 1F** **)**.

### Pathological Alterations and Lipid Deposition in Diabetic Nephropathy Patients

**Figure 2:**
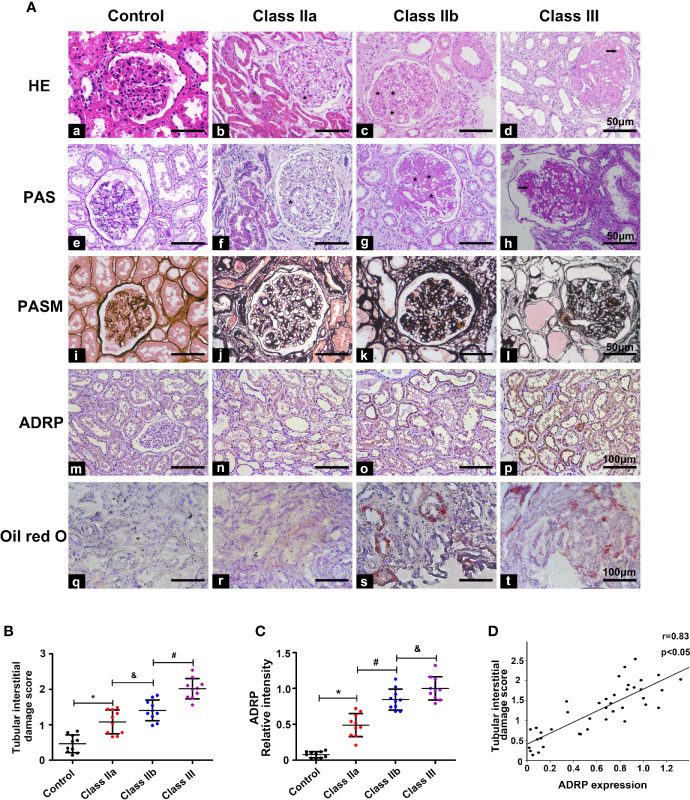
Pathological changes and the correlation between tubulointerstitial damage and lipid deposition. One-way ANOVA was used to compare the differences among the groups, and the correlation of the two numerical variables was tested by Pearson’s analysis. **(A)** HE, PAS, Masson, IHC, and oil red O staining were used to detect pathological changes and ADRP expression in the kidney of the different groups. **(B)** The tubular interstitial damage score was used to assess the degree of tubular injury. **(C)** Scatter diagram representing the levels of ADRP. **(D)** Correlation analysis between the expression of ADRP and the tubular interstitial damage score. The asterisk (*) represents the notable mesangial expansion, and the arrows represent typical Kimmelstiel-Wilson lesions. Experiments were performed at least three times for each donor, with a minimum of five random fields taken per condition. Values are the mean±SD. *p < 0.05 compared with the control group; ^#^p < 0.05 compared with the class IIa group; ^&^p < 0.05 compared with the class IIb group.

HE, PAS, and PASM staining were used to evaluate the pathological lesion changes in the kidneys of the DN patients, including the glomeruli and tubulointerstitium. HE staining showed that the samples from class IIa and IIb had significantly exfoliated tubule cells, while the PAS and PASM staining showed notable mesangial expansion, and typical Kimmelstiel-Wilson lesions were observed in the class III renal tissues of DN patients (**Figure 2A**). Moreover, we used the IFTA scoring system to assess the severity of tubulointerstitial injury, as previously described (17). Statistical analysis showed that the IFTA scores were higher in the class IIa and class IIb groups than in the control group, and were even higher in the class III group (**Figure 2B**). Meanwhile, the expression of ADRP (the marker protein of lipid droplets) and oil red O staining from renal biopsies of DN patients were used to evaluate the lipid deposition in DN. Compared with the control group, a notable increase in ADRP levels and the yellow area of oil red O staining were observed in the kidneys of the class IIa and IIb groups, and this increase was further aggravated in the class III group (**Figures 2A, C**). In addition, correlation analyses revealed a significant positive correlation between the expression of ADRP and the tubular interstitial damage score (**Figure 2D**, r = 0.83, p < 0.05).

### Relevance of Mitochondria-associated Endoplasmic Reticulum Membranes to Lipid Deposition in the Kidney of Diabetic Nephropathy

**Figure 3:**
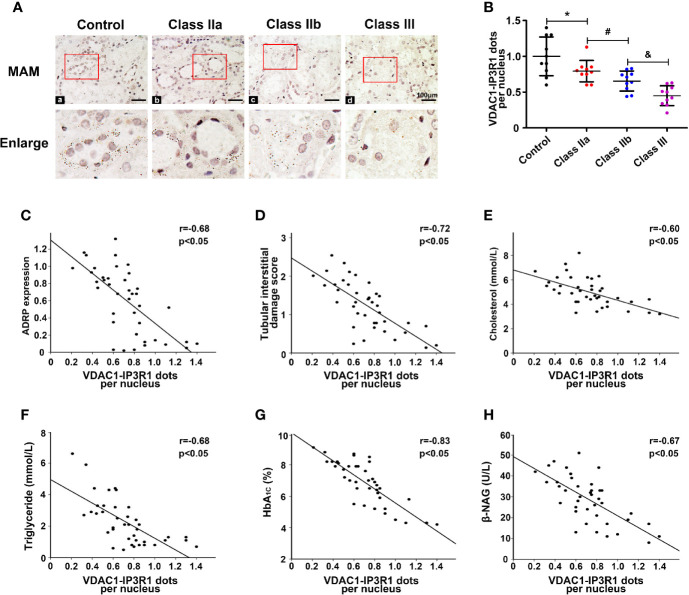
Disruption of MAM coupling in DN patients and the correlation between the MAMs, lipid deposition, and kidney injury. One-way ANOVA was used to compare the differences among groups, and the correlation of two numerical variables was tested by Pearson’s analysis. **(A)** A significant decrease in MAMs was observed in the kidneys of the DN groups compared with the control. **(B)** Scatter diagram representing the level of the MAMs. Correlation analysis between the MAMs and the expression of ADRP **(C)**, tubular interstitial damage score **(D)**, CHOL level **(E)**, TG level **(F)**, HbA1c level **(G)**, and β-NAG level **(H)**. Experiments were performed at least three times for each donor, with a minimum of five random fields taken per condition. Values are the mean±SD. *p < 0.05 compared with the control group; ^#^p < 0.05 compared with the class IIa group; ^&^p < 0.05 compared with the class IIb group.

To confirm the relationship between the MAMs and lipid deposition in the kidneys of DN patients, *in situ* PLA was used to detect the number of MAMs by immunostaining of the ER protein (IP3R1) and mitochondrial protein (VDAC1). MAM-associated staining using VDAC1/IP3R1 showed a significant decrease in the MAMs in tubular cells of the kidney in DN compared to the control (**Figures 3A** **)**, and there was no positive signal when VDAC1 or IP3R1 was used alone (**Figure Supplementary 1**). Similar results were seen in the different DN groups. There were markedly decreased MAMs in the class IIb group compared to the class IIa group, and the MAMs were further reduced in the class III group (**Figures 3A, B** **)**. Similar results were also observed when the integrity of the MAMs was also detected by TEM (**Figure Supplementary 2**). Correlation analyses revealed that there was a strong negative correlation between the MAMs and the expression of ADRP (**Figure 3C**, r = −0.68, p < 0.05), the tubular interstitial damage score (**Figure 3D**, r = −0.72, p < 0.05), the levels of cholesterol (**Figure 3E**, r = −0.60, p < 0.05), TG (**Figure 3F**, r = −0.68, p < 0.05), HbA1c (**Figure 3G**, r = −0.83, p < 0.05), and β-NAG (**Figure 3H**, r = −0.67, p < 0.05).

### The Expression of Mitochondria-associated Endoplasmic Reticulum Membrane-*C*ontrol Proteins Was Decreased in the Diabetic Nephropathy Patients and Had a Negative Correlation With ADRP

**Figure 4:**
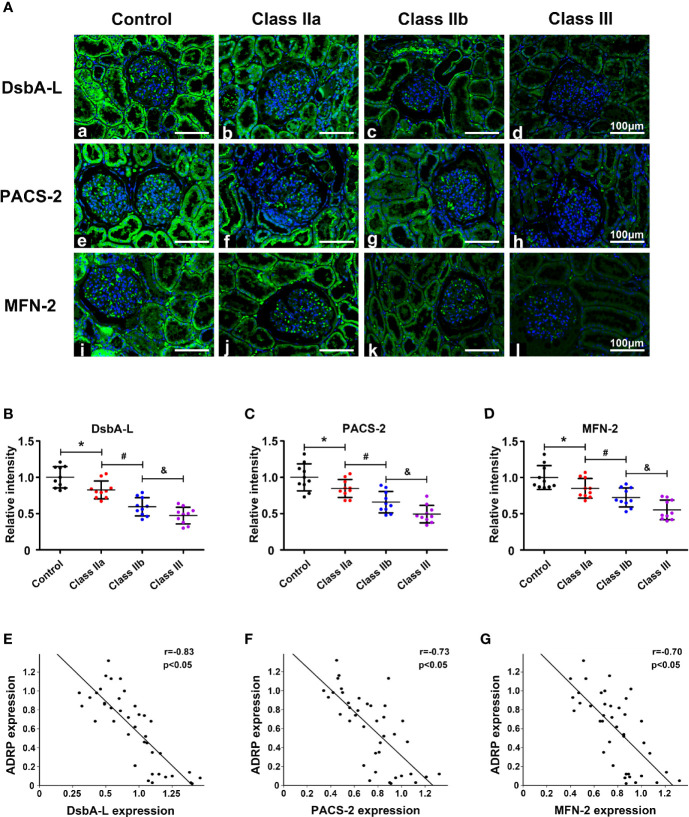
The expression of MAM-control proteins (DsbA-L, PACS-2, and MFN-2) and the correlation between these proteins and ADRP. One-way ANOVA was used to compare the differences among groups, and the correlation of the two numerical variables was tested by Pearson’s analysis. **(A)** IHC staining revealed a notably reduced expression of DsbA-L, PACS-2, and MFN-2 in the kidneys of DN patients. Scatter diagram representing the levels of DsbA-L **(B)**, PACS-2 **(C)**, and MFN-2 **(D)**. **(E)** Correlation analysis between the expression of DsbA-L and ADRP. **(F)** Correlation analysis between the expression of PACS-2 and ADRP. **(G)** Correlation analysis between the expression of MFN-2 and ADRP. Experiments were performed at least three times for each donor, with a minimum of five random fields taken per condition. Values are the mean±SD. *p < 0.05 compared with the control group; ^#^p < 0.05 compared with the class IIa group; ^&^p < 0.05 compared with the class IIb group.

To further confirm the correlation between the MAMs and lipid deposition, we selected three MAM-control proteins DsbA-L, PACS-2, and MFN-2, which have been shown to control the formation of MAMs and IHC staining was used to observe their expression in renal tissue. IHC revealed substantially decreased expression of DsbA-L in the kidneys of the class IIa and IIb groups compared with the control group, while they were further decreased in the class III DN group (**Figures 4Aa–d, B**). Similar results were observed for the expression of PACS-2 (**Figures 4Ae–h, C**) and MFN-2 (**Figures 4Ai-l, D**). Correlation analyses revealed that there was a significant negative correlation between the expression of DsbA-L (**Figure 4E**, r = −0.83, p < 0.05), PACS-2 (**Figure 4F**, r = −0.73, p < 0.05), MFN-2 (**Figure 4G**, r = −0.70, p < 0.05), and ADRP in the kidney.

## Discussion

Disorders of lipid metabolism and renal ectopic fat deposition are considered to be important pathological changes in DN, and they are also pathways of hyperglycemia-induced kidney injury (7, 19). MAMs are the coupling between the ER and mitochondria, both of which are critical structures involved in lipid metabolism in cells (13). However, the relationship between the integrity of MAMs and the clinical characteristics of patients with DN has not been well clarified. We demonstrated some meaningful findings in this study. First, we observed that lipid deposition in the kidney was notably increased in DN patients compared with controls and it was closely related to renal injury. Then, *in situ* PLA was used to detect the integrity of the MAMs. The integrity of the MAMs was found to be destroyed in the kidneys of the DN patients, and there was a strong negative correlation between the MAMs and the serum lipid levels, renal lipid deposition, and kidney injury. Finally, to further confirm the relationship between the MAMs and lipid deposition in the kidney, we observed decreased expression of MAM-control proteins (DsbA-L, PACS-2, and MFN-2) in different stages of DN, and they were associated with lipid deposition and renal damage. These findings suggested that the destruction of the MAM integrity may contribute to renal lipid deposition and the progression of kidney damage in DN.

The role of dyslipidemia and renal lipid deposition in the development of DN has been widely studied. A study of DCCT/EDIC demonstrated that lower levels of LDL-C and TG were associated with a lower risk of progression from moderate proteinuria to severe proteinuria or end-stage renal disease (ESRD) (20). In addition, fenofibrate, a commonly used drug to reduce serum cholesterol levels in the clinic, has also been shown to delay the course of diabetes complications (21). Low LDL-c and TG levels are also associated with the improvement from moderate proteinuria to normoalbuminuria in diabetic patients (22, 23). Furthermore, the presence of lipid disorders in diabetes can lead to lipid deposition in the kidney. Lipid deposition in the kidney will damage the fatty acid β-oxidation of renal cells and subsequently increase the expression of fibrotic factors (24). In addition, the accumulation of lipids in renal epithelial tubular cells also causes the increased production and release of reactive oxygen species and inflammatory cytokines, which are closely related to the development of DN (25). However, the mechanism of renal lipid deposition has not been well explored. Consistent with previous studies, we also observed that dyslipidemia and renal lipid deposition were positively correlated with pathological changes in the kidney in DN patients. We also observed for the first time that ER-mitochondrial coupling, also known as MAMs, was significantly reduced in the renal tissue of DN patients and was negatively correlated with lipid levels and indicators of kidney damage, which means that MAMs may be involved in the renal injury induced by lipids in DN.

As the bridge between the ER and mitochondria (the most active organelles in the cell), the MAM plays an irreplaceable role in maintaining cell homeostasis, and participation in lipid metabolism is also one of the main functions of MAMs. Since the 1990s, when Vance extracted the ER subdomains and named them “MAMs,” an increasing number of lipid-metabolizing enzymes have been found. The key enzymes involved in phospholipid, triglyceride, and cholesterol metabolism, such as DGAT2, PSS, and ACAT, are important components of MAMs (12), and MAMs are also thought to be involved in the formation of fat droplets (26). In general, MAMs act as platforms for lipid synthesis and transport between the ER and mitochondria. In a previous study, we showed that the integrity of the MAMs in the kidneys of diabetic mice and in HK-2 cells treated with high glucose was destroyed, thus promoting apoptosis (16). Other groups also found that in the presence of palmitic acid *in vitro*, the MAMs were destroyed and this caused insulin resistance, while restoring the integrity of MAMs can improve insulin sensitivity (14, 27), implying that in the case of metabolic diseases, the disruption of MAMs can further aggravate metabolic disorders. In this study, we used *in situ* PLA staining to directly detect MAMs in the kidney. We observed that the integrity of MAMs was decreased in the kidneys of DN patients, and correlation analysis showed that the integrity of MAMs was closely correlated with serum lipid levels, renal lipid deposition, and tubular interstitial damage. This suggests that MAMs play a potential role in the process of renal lipid deposition and renal injury in patients with DN.

To further verify our hypothesis that the changes in MAMs resulted in renal lipid deposition in DN, we selected three MAM-control proteins, DsbA-L, PACS-2, and MFN-2, which have been proven by us and other groups to control the formation of MAMs (16, 28–30). We noted that the expression of DsbA-L, PACS-2, and MFN-2 was decreased in the kidneys of the DN patients compared to those of the controls. They were closely correlated with the expression of ADRP and pathological injury of the kidney. Interestingly, the destruction of MAM integrity and increased lipid deposition and apoptosis were observed in HK-2 cells treated with high glucose, while these phenomena were alleviated once MAM integrity was restored by overexpression of DsbA-L (**Figure Supplementary 3A–D**). These data further verified our hypothesis that the destruction of MAM integrity in kidneys of DN patients results in renal lipid deposition and renal damage.

Although there are many questions that need to be answered, some key ones are: what is the molecular mechanism of renal lipid deposition caused by the destruction of MAM integrity in the kidney? Does the restoration of the MAM integrity improve renal lipid deposition in DN? Experiments *in vivo* and *in vitro* are needed to verify this. Here, we demonstrated that the integrity of the MAMs was reduced in the kidneys of DN patients and that this was related to renal lipid deposition. We believe this work explains the pathogenesis of diabetic nephropathy from a new perspective.

## Data Availability Statement

The original contributions presented in the study are included in the article/**Supplementary Material**. Further inquiries can be directed to the corresponding author.

## Ethics Statement

The studies involving human participants were reviewed and approved by the medical ethics committee of The Second Xiangya Hospital, Central South University. The patients/participants provided their written informed consent to participate in this study.

## Author Contributions

MY and YH designed the study, analyzed the data, interpreted the results, and drafted the manuscript. YX, LW, XX, CL, NJ, and SL contributed to the data collection and manuscript revision. HZ, JY, and XZ provided support for interpreting the results and revising the manuscript. LS is the corresponding author and was involved in the study design, data interpretation, and manuscript revision. All authors contributed to the article and approved the submitted version.

## Funding

This work was supported by grants from the National Natural Science Foundation of China (81730018) and the National Key R&D Program of China (2018YFC1314002).

## Conflict of Interest

The authors declare that the research was conducted in the absence of any commercial or financial relationships that could be construed as a potential conflict of interest.

## Supplementary Material

Supplementary Figure 1 The expression of VDAC1, Fis1, and Drp1 in DN patients. **(A)** *In situ* PLA staining using VDAC1 or IP3R1 alone or using VDAC1 and IP3R together. **(B)** The expression of VDAC1, Fis1, and Drp1. **(C–E)** Scatter diagram representing the levels of VDAC1, Fis1, and Drp1. Experiments were performed at least three times for each donor, with a minimum of five random fields taken per condition. Values are the mean±SD. *p < 0.05 compared with the control group; ^#^p < 0.05 compared with the class IIa group.

Supplementary Figure 2 The integrity of the MAMs detected by TEM.

Supplementary Figure 3 Altered MAM integrity and lipid deposition in HK-2 cells. **(A)** Confocal image with Mito-Tracker and ER-Tracker staining showing MAM integrity in the HK-2 cells. **(B)** Quantitative analysis of MAMs by a graph. **(C)** Oil red O staining in the different HK-2 groups. **(D)** WB analysis of cleaved caspase 3 in the HK-2 cells. The values are the mean±SD. *p < 0.05 compared with the LG group; ^#^p < 0.05 compared with the HG group.
