# Supplementary figures and images for: MAMs Protect Against Ectopic Fat Deposition and Lipid-Related Kidney Damage in DN Patients
Source: Front Endocrinol (Lausanne). 2021 Feb 19;12:609580. doi: 10.3389/fendo.2021.609580 (PMC7933555; doi:10.3389/fendo.2021.609580)

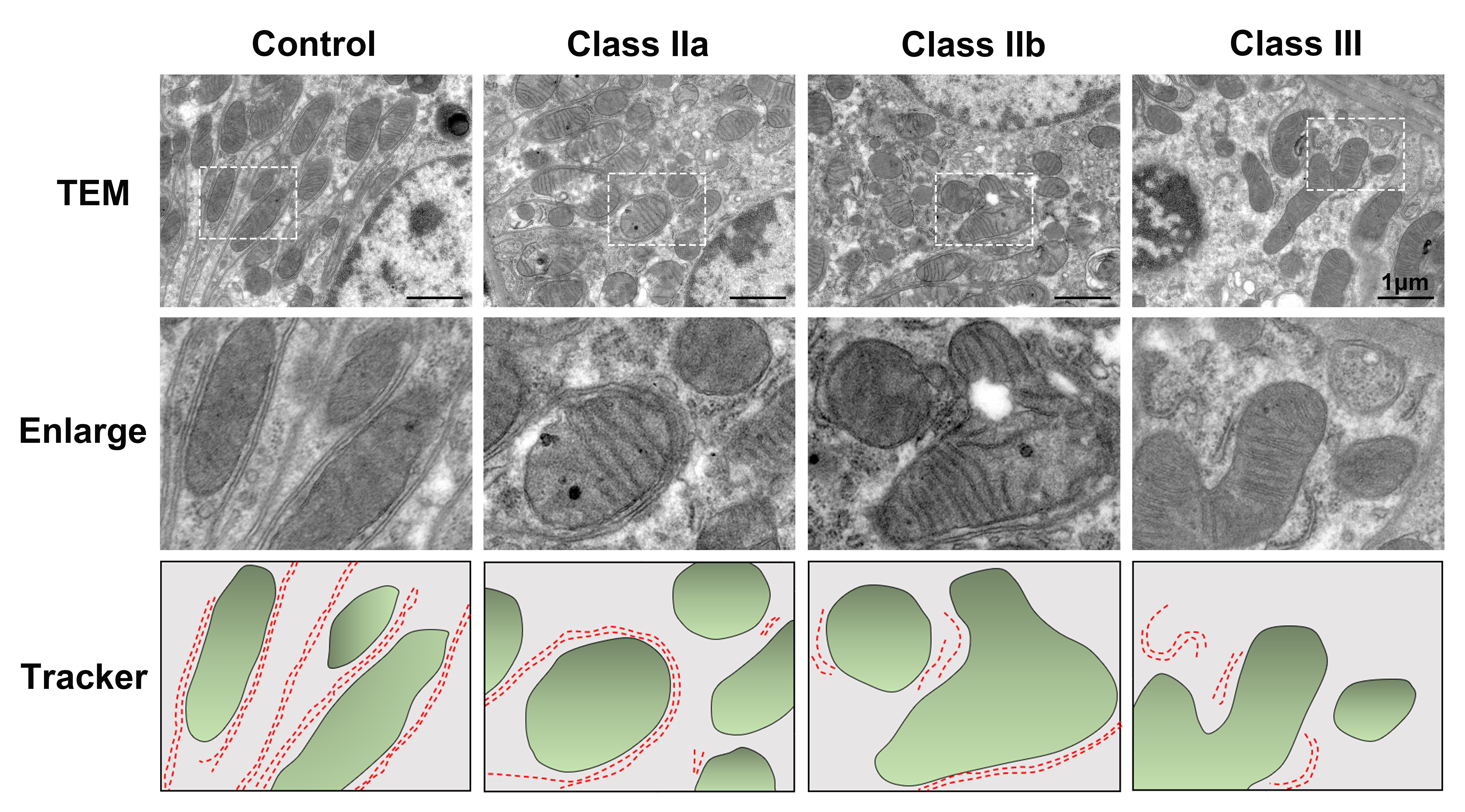

Supplement: Supplementary Figure 2 — The integrity of the MAMs detected by TEM. [file Image_2.jpeg]
